# Correlations among lymphocyte count, white matter hyperintensity and brain atrophy in patients with ischemic stroke

**DOI:** 10.3389/fnagi.2024.1492078

**Published:** 2025-01-08

**Authors:** Chenchen Liu, Dai Shi, Xiaoqiong Ni, Shoujiang You, Xiaofen Wu, Sheng Zhuang, Wu Cai, Liang Xu

**Affiliations:** ^1^Department of Radiology, Clinical Research Center of Neurological Disease, The Second Affiliated Hospital of Soochow University, Suzhou, China; ^2^Department of Neurology, The Second Affiliated Hospital of Soochow University, Suzhou, China; ^3^Department of Neurosurgery, The Second Affiliated Hospital of Soochow University, Suzhou, China

**Keywords:** lymphocyte count, white matter hyperintensity, brain atrophy, ischemic stroke, association

## Abstract

**Background:**

White matter hyperintensity (WMH) and brain atrophy, as imaging marker of cerebral small-vessel diseases (CSVD), have a high prevalence and strong prognostic value in stroke. We aimed to explore the association between lymphocyte count, a maker of inflammation, and WMH and brain atrophy in patients with acute ischemic stroke (AIS).

**Methods:**

A total of 727 AIS patients with lymphocyte count and brain magnetic resonance imaging data were enrolled. Participants were divided into four groups according to the quartiles of baseline lymphocyte counts. WMH is frequently divided into periventricular hyperintensity (PVH) and deep white matter hyperintensity (DWMH). WMH was defined as Fazekas scale score ≥ 3; PVH was defined as periventricular Fazekas scale ≥2; DWMH was defined as deep Fazekas scale ≥2. Brain atrophy was defined as global cortical atrophy score ≥ 1. Multivariable logistic regression models were used to assess the association between lymphocyte count and WMH and brain atrophy.

**Results:**

Among 727 AIS, 517 (71.1%), 442 (60.8%), 459 (63.1%), 583 (80.2%) had WMH, PVH, DWMH and brain atrophy, respectively. After adjustment for potential covariates, the highest quartiles of lymphocyte counts were significantly associated with lower risk of WMH (adjusted odds ratio [aOR] 0.57, 95% confidence intervals [CI] 0.32–0.99), PVH (aOR 0.52, 95% CI 0.31–0.87), DWMH (aOR 0.53 95% CI 0.32–0.90) as well as brain atrophy (aOR 0.46, 95% CI 0.23–0.92) compared with the lowest quartiles of lymphocyte counts, respectively. Furthermore, a notable inverse association was observed between continuous lymphocyte counts and WMH, PVH, DWMH, and brain atrophy. Additionally, we found that the inverse association between baseline lymphocyte count and WMH was significant only in individuals with mild stroke.

**Conclusion:**

In patients with AIS, there was an independent and inverse association between the baseline lymphocyte count and both WMH and brain atrophy.

## 1 Introduction

Cerebral small vessel disease (CSVD) is the most common pathological neurological process and can present as stroke, cognitive decline, neurobehavioral symptoms, or functional impairment (Wardlaw et al., 2019). Neuroimaging features of CSVD include recent small subcortical infarcts, lacunes, white matter hyperintensities (WMH), perivascular spaces, microbleeds, and brain atrophy (Wardlaw et al., 2013). WMH and brain atrophy, commonly found in stroke patients, are associated with cognitive impairment, recurrence of stroke, and worse outcomes after stroke and are considered to be the key magnetic resonance imaging (MRI) makers of CSVD (Wardlaw et al., 2013; Li et al., 2022; De-Guio et al., 2019; Georgakis et al., 2019; Kaginele et al., 2021; Debette et al., 2019). However, the mechanism of CSVD is still incompletely clarified and inflammation may play an important role. Previous research has demonstrated a strong link between peripheral blood markers and CSVD (Li et al., 2022; Kong et al., 2024; Li et al., 2024; Wan et al., 2023; Zhang et al., 2022; Evans et al., 2021).

Lymphocytes, a common marker in routine blood tests, play a crucial role in inflammation and the immune response. Lymphocyte depletion may indicate a chronic inflammatory condition (Nam et al., 2022). Observational studies have shown that lymphopenia is associated with an increased risk of cardiovascular disease (Núñez et al., 2011; Rudiger et al., 2006), cancer (Ray-Coquard et al., 2009), infectious disease (Warny et al., 2018), and systemic autoimmune disease (Rivero et al., 1978). However, population-based evidence on the relationship between baseline lymphocyte count and CSVD, particularly, WMH and brain atrophy, has not been clearly established, especially in the setting of ischemic stroke. The aim of the present study was to explore the relationship between baseline lymphocyte count and WMH and brain atrophy in patients with acute ischemic stroke (AIS), thereby gaining insights into the mechanisms underlying the pathophysiology of CSVD.

## 2 Materials and methods

### 2.1 Study participants

We consecutively enrolled patients with ischemic stroke at the Second Affiliated Hospital of Soochow University from January 2019 to February 2022. The diagnosis of ischemic stroke was made according to World Health Organization-defined criteria based on patient history, clinical characteristics, and neuroimaging (brain MRI or computed tomography [CT] scans). The exclusion criteria were as follows: (i) lack of brain MRI data, largely due to severe neurological deficits in AIS patients, which rendered them unable to cooperate with the MRI examination; and (ii) lack of lymphocyte count. Finally, 727 ischemic stroke patients with available brain MRI and lymphocyte count data were included in this analysis.

### 2.2 Data collection

Data on demographic characteristics, clinical features, medical history, medication history, and imaging data were collected at the time of enrollment. Current smoking status was defined as at least smoking one cigarette per day for 1 year or more. Alcohol consumption was defined as consuming at least one alcoholic drink per day over the past year. Stroke severity was evaluated by qualified neurologists using the National Institutes of Health Stroke Scale (NIHSS) at admission (Brott et al., 1989). Trial of Org 1, 0172 in Acute Stroke Treatment (TOAST) classification was used to classify the subtypes of AIS (Pickering et al., 2005). Blood samples were collected and tested within 24 h after hospital admission for all participating patients. Lymphocyte counts, as a marker of routine blood tests were measured by flow cytometry (XE-2100, SYSMES, Kobe, Japan).

### 2.3 WMH and brain atrophy scoring

All patients underwent head MRI with T1-weighted (T1WI), T2-weighted (T2WI), and T2-weighted fluid-attenuated inversion recovery (T2WI-FLAIR) sequences within the first 5 days after admission. WMH of presumed vascular origin was defined as lesions with hyperintensities on T2WI and FLAIR imaging and hypointensities on T1WI, and they were distinguished as periventricular hyperintensity (PVH) and deep white matter hyperintensity (DWMH) according to the Fazekas scale (Wardlaw et al., 2013; Fazekas et al., 1987). WMH was defined as Fazekas scale score ≥ 3; PVH was defined as periventricular Fazekas scale ≥2; DWMH was defined as deep Fazekas scale ≥2. Brain atrophy was defined as a lower brain volume that is not related to a specific macroscopic focal injury such as trauma or infarction (Wardlaw et al., 2013). The evaluation of global cortical atrophy (GCA) was based on axial T1 sequences and if not available on fluid-attenuated inversion recovery sequences. Brain atrophy was defined as GCA score ≥ 1. Imaging data were independently read and assessed by two experienced radiologists blinded to the clinical data. All disagreements were resolved by consensus.

### 2.4 Statistical analysis

Patients in this study were divided into four groups according to the quartiles of baseline lymphocyte count: Q1 < 1.2 × 10^9^/L, Q2: 1.2–1.6 × 10^9^/L, Q3: 1.6–2.1 × 10^9^/L, Q4: ≥ 2.1 × 10^9^/L. Continuous variables were expressed as mean ± standard deviation (SD) or median (interquartile range [IQR]) and were compared across the four groups using the analysis of variance or Wilcoxon rank-sum test. Categorical variables were expressed as frequency (%) and were compared among the four groups using the chi-square test.

Multivariate logistic regression was used to estimate the risk of WMH, PVH, DWMH and brain atrophy among quartiles of baseline lymphocyte count. Odds ratios (ORs) and 95% confidence interval (CIs) were calculated for each group with the lowest quartile (Q1) as reference. Linear trends were assessed by treating categorical variables as ordinal variables in the models. Potential confounders selected from previous knowledge and the difference across the quartiles of baseline lymphocyte count, such as sex, age (<69 vs. ≥69 years old, median), current smoking and alcohol drinking, systolic blood pressure (BP), fasting plasma glucose, medical history (hypertension, diabetes mellitus, coronary heart disease, atrial fibrillation, ischemic stroke, intracranial hemorrhage), antihypertension treatment, antiglycemia treatment, fibrinogen, neutrophil count were included in the multivariate model (Carmichael et al., 2019; Jiang et al., 2022; You et al., 2018).

To assess the robustness of the association between baseline lymphocyte count and WMH, PVH, DWMH and brain atrophy, we also performed additional analysis treating baseline lymphocyte count as a continuous variable. To assess the generality of the association between baseline lymphocyte count and WMH and brain atrophy, subgroup analyses were conducted in multivariable-adjusted models stratified by baseline NIHSS (≤3 vs. >3, mild stroke criteria), stroke subtype (Large-artery atherosclerosis [LAA] vs. Non-LAA). Receiver operating characteristic analyses were used to assess the predictive performance of baseline lymphocyte count on WMH, PVH, DWMH and brain atrophy. All *p* values were two-tailed, and a significance level of 0.05 was used. All analyses were conducted using the SPSS software (version 16.0).

## 3 Results

### 3.1 Baseline characteristics

A total of 727 acute ischemic stroke patients with available data (mean [SD] age, 66.8 [12.8] years; 467 [64.2%] male) were included. Baseline characteristics of the patients according to lymphocyte count quartiles were shown in Table 1. In comparison to participants with lower lymphocyte count, those with higher lymphocyte count were more likely to be younger; to have higher total cholesterol, low-density lipoprotein cholesterol and lower baseline NIHSS score. Patients with higher lymphocyte counts also exhibited a reduced prevalence of a history of ischemic stroke and a decreased occurrence of WMH, including PVH, DWMH, and brain atrophy compared to those with lower lymphocyte counts.

**Table 1:** Baseline characteristics of 727 acute ischemic stroke patients according to plasma lymphocyte count quartiles.

Lymphocyte count, 10^9^/L					
Characteristics ^a^	Q1 <1.2	Q2 1.2–1.6	Q3 1.6–2.1	Q4 ≥2.1	*p*-value
Number of subjects	174	173	190	190	
Demographics					
Age, y	69.4 ± 12.0	69.2 ± 11.8	65.2 ± 12.5	64.0 ± 13.8	<0.001
Male sex	117 (67.2)	118 (68.2)	108 (56.8)	124 (65.3)	0.090
Current cigarette smoking status	53 (30.5)	69 (39.9)	71 (37.4)	74 (38.9)	0.249
Current alcohol consumption	41 (23.6)	54 (31.2)	50 (26.3)	47 (24.7)	0.383
Clinical features					
Time from onset to hospital, h	12.0 (7.0–23.0)	12.0 (7.0–48.0)	12.0 (7.0–48.0)	12.0 (7.0–48.0)	0.714
Baseline systolic BP, mm Hg	149.3 ± 26.2	151.1 ± 21.9	152.4 ± 24.6	151.4 ± 23.3	0.673
Baseline diastolic BP, mm Hg	83.2 ± 14.6	82.7 ± 12.9	84.7 ± 13.7	84.3 ± 14.0	0.450
Neutrophil count, 10^3^/μL	4.6 (3.7–7.2)	4.4 (3.4–5.5)	4.6 (3.5–5.8)	4.7 (3.8–6.1)	0.288
TC, mmol/L	4.2 (3.8–5.0)	4.4 (4.0–5.0)	4.8 (3.9–5.3)	4.7 (4.1–5.3)	<0.001
LDL-C, mmol/L	2.5 (1.9–3.1)	2.6 (2.1–3.2)	3.0 (2.1–3.6)	2.9 (2.4–3.4)	<0.001
HDL-C, mmol/L	1.1 (0.9–1.3)	1.1 (0.9–1.4)	1.0 (0.9–1.3)	1.0 (0.9–1.3)	0.242
FIB	3.2 (2.6–3.8)	3.1 (2.6–3.6)	3.2 (2.6–3.6)	3.1 (2.6–3.6)	0.810
FPG, mmol/L	5.6 (5.0–6.8)	5.4 (5.0–6.6)	5.7 (5.0–7.3)	5.6 (5.1–6.9)	0.321
Baseline NIHSS score	3.0 (1.0–7.0)	2.0 (1.0–5.0)	2.0 (1.0–4.0)	2.0 (1.0–3.0)	<0.001
Medical history					
History of hypertension	139 (79.9)	136 (78.6)	140 (73.7)	142 (74.7)	0.439
History of diabetes mellitus	59 (33.9)	58 (33.5)	65 (34.2)	66 (34.7)	0.996
History of coronary heart disease	6 (3.4)	9 (5.2)	6 (3.2)	4 (2.1)	0.452
History of atrial fibrillation	30 (17.2)	15 (8.7)	23 (12.1)	20 (10.5)	0.083
History of ischemic stroke	55 (31.6)	47 (27.2)	47 (24.7)	34 (17.9)	0.023
History of ICH	6 (3.4)	3 (1.7)	1 (0.5)	3 (1.6)	0.212
Medication history					
Antihypertensive therapy	103 (59.2)	105 (60.7)	107 (56.3)	103 (54.2)	0.598
Antiplatelet therapy	35 (20.1)	29 (16.8)	23 (12.1)	22 (11.6)	0.072
Statin therapy	20 (11.5)	24 (13.9)	17 (8.9)	16 (8.4)	0.307
Antiglycemia treatment	34 (19.5)	39 (22.5)	41 (21.6)	42 (22.1)	0.908
TOAST classification					0.111
Large-artery atherosclerosis	106 (60.9)	105 (60.7)	124 (65.3)	116 (61.1)	
Cardioembolism	31 (17.8)	16 (9.2)	24 (12.6)	22 (11.6)	
Small-vessel occlusion	25 (14.4)	41 (23.7)	33 (17.4)	43 (22.6)	
Stroke of other determined etiology	12 (6.9)	7 (4.0)	7 (3.7)	7 (3.7)	
Stroke of undetermined etiology	0 (0.0)	4 (2.3)	2 (1.1)	2 (1.1)	
WMH	137 (78.7)	129 (74.6)	132 (69.5)	119 (62.6)	0.005
PVH	122 (70.1)	116 (67.1)	107 (56.3)	97 (51.1)	<0.001
DWMH	125 (71.8)	113 (65.3)	122 (64.2)	99 (52.1)	0.001
Brain atrophy	152 (87.4)	150 (86.7)	142 (74.7)	139 (73.2)	<0.001

^*^Continuous variables are expressed as mean ± standard deviation or as median (interquartile range). Categorical variables are expressed as frequency (percent).

BP, blood pressure; TC, total cholesterol; LDL-C, low-density lipoprotein cholesterol; HDL-C, high-density lipoprotein cholesterol; FPG, fasting plasma glucose; NIHSS, National Institutes of Health Stroke Scale; WMH, white matter hyperintensities; PVH, periventricular hyperintensity; DWMH, deep white matter hyperintensity; Q, quartile.

### 3.2 Lymphocyte count with WMH, PVH and DWMH

**Figure 1:**
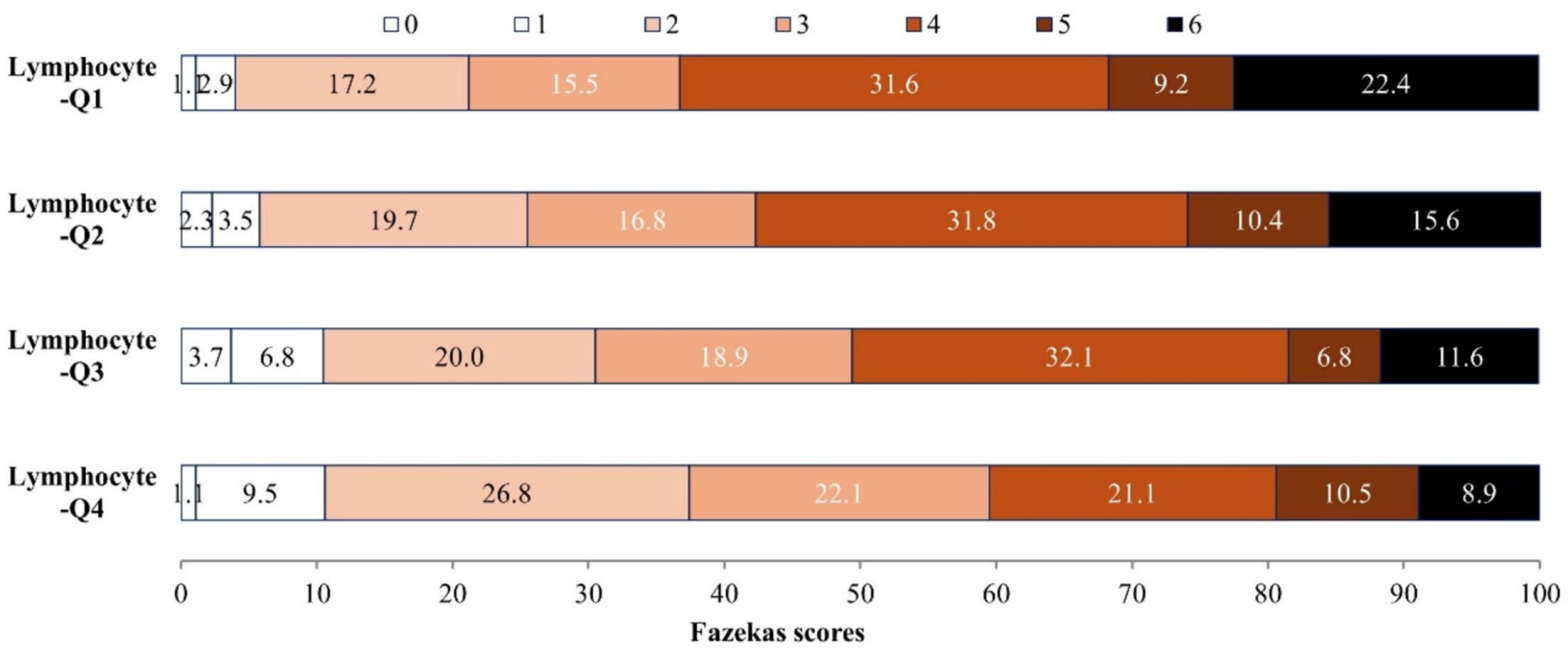
Associations of lymphocyte count with severity of WMH.

Among 727 AIS patients, there are 517 (71.1%), 442 (60.8%), 459 (63.1%) patients presented with WMH, PVH and DWMH, respectively. The distribution of Fazekas scale score across the quartiles of lymphocyte count are shown in Figure 1, patients with higher lymphocyte count exhibit significantly lower Fazekas scale scores (*p* < 0.001). In the unadjusted logistic regression model, the ORs of WMH, PVH and DWMH were significantly lower among study participants with admission lymphocyte count in the highest quartile (Q4 ≥ 2.1*10^9^/L) compared with those in the lowest quartile (Q1 < 1.2* 10^9^/L) (Table 2). After adjusting for age, sex, systolic BP, fasting plasma glucose, medical history, and other covariates, compared with the lowest quartile, the ORs (95% CIs) for the highest quartile of lymphocyte counts were 0.57 (0.32–0.99) for WMH, 0.52 (0.31–0.87) for PVH, and 0.53 (0.32–0.90) for DWMH, respectively (Table 2). Furthermore, a higher level of lymphocyte count was still significantly associated with decreased odds of WMH (OR 0.75, 95% CI 0.58–0.97), PVH (OR 0.76, 95% CI 0.59–0.97) and DWMH (OR 0.72, 95% CI 0.56–0.92), when considering lymphocyte counts as continue variable (Table 2). The Area under the curve (AUCs) and *p*-values for baseline lymphocyte count in predicting WMH, PVH and DWMH were 0.591 (95% CI: 0.545–0.636, *p* < 0.001), 0.592 (95% CI: 0.550–0.634, *p* < 0.001), and 0.594 (95% CI, 0.551–0.636, *p* < 0.001) (Supplementary Figure S1).

**Table 2:** Associations of lymphocyte count with WMH, PVH and DWMH.

Lymphocyte count, 10^9^/L						Continuous of lymphocyte	
	Q1: <1.2	Q2: 1.2–1.6	Q3: 1.6–2.1	Q4: ≥2.1	*p* trend		
Median	0.9	1.3	1.8	2.4			
WMH							
Events, n (%)	137 (78.7)	129 (74.6)	132 (69.5)	119 (62.6)			
Model 1	1.00	0.79 (0.48–1.30)	0.62 (0.38–0.99)	0.45 (0.28–0.72)	<0.001	0.66 (0.53–0.81)	
Model 2	1.00	0.79 (0.46–1.34)	0.70 (0.42–1.16)	0.52 (0.32–0.86)	0.009	0.71 (0.56–0.89)	
Model 3	1.00	0.80 (0.44–1.47)	0.81 (0.45–1.44)	0.57 (0.32–0.99)	0.052	0.75 (0.58–0.97)	
PVH							
Events, n (%)	122 (70.1)	116 (67.1)	107 (56.3)	97 (51.1)			
Model 1	1.00	0.87 (0.55–1.37)	0.55 (0.36–0.85)	0.45 (0.29–0.68)	<0.001	0.67 (0.55–0.82)	
Model 2	1.00	0.87 (0.53–1.42)	0.64 (0.40–1.03)	0.52 (0.33–0.83)	0.003	0.74 (0.60–0.93)	
Model 3	1.00	0.82 (0.48–1.42)	0.65 (0.38–1.10)	0.52 (0.31–0.87)	0.008	0.76 (0.59–0.97)	
DWMH							
Events, n (%)	125 (71.8)	113 (65.3)	122 (64.2)	99 (52.1)			
Model 1	1.00	0.74 (0.47–1.16)	0.70 (0.45–1.10)	0.43 (0.28–0.66)	<0.001	0.63 (0.51–0.78)	
Model 2	1.00	0.73 (0.45–1.17)	0.81 (0.50–1.29)	0.48 (0.31–0.77)	0.004	0.68 (0.54–0.84)	
Model 3	1.00	0.80 (0.46–1.38)	0.93 (0.54–1.58)	0.53 (0.32–0.90)	0.028	0.72 (0.56–0.92)	

WMH, white matter hyperintensities; PVH, periventricular hyperintensity; DWMH, deep white matter hyperintensity.

Model 1: unadjusted model.

Model 2: adjusted for age (<69 vs. ≥ 69 years old), sex, current smoking and alcohol drinking.

Model 3: adjusted for sex, age (<69 vs. ≥ 69 years old), current smoking and alcohol drinking, systolic BP, fasting plasma glucose, medical history (hypertension, diabetes mellitus, coronary heart disease, atrial fibrillation, ischemic stroke, intracranial hemorrhage), antihypertension treatment, antiglycemia treatment, fibrinogen, neutrophil count.

WMH defined as Fazekas scale score ≥ 3; PVH, defined as periventricular hyperintensity score based on Fazekas scale ≥ 2; DWMH defined as deep white matter hyperintensity based on Fazekas scale ≥ 2.

### 3.3 Lymphocyte count with brain atrophy

**Figure 2:**
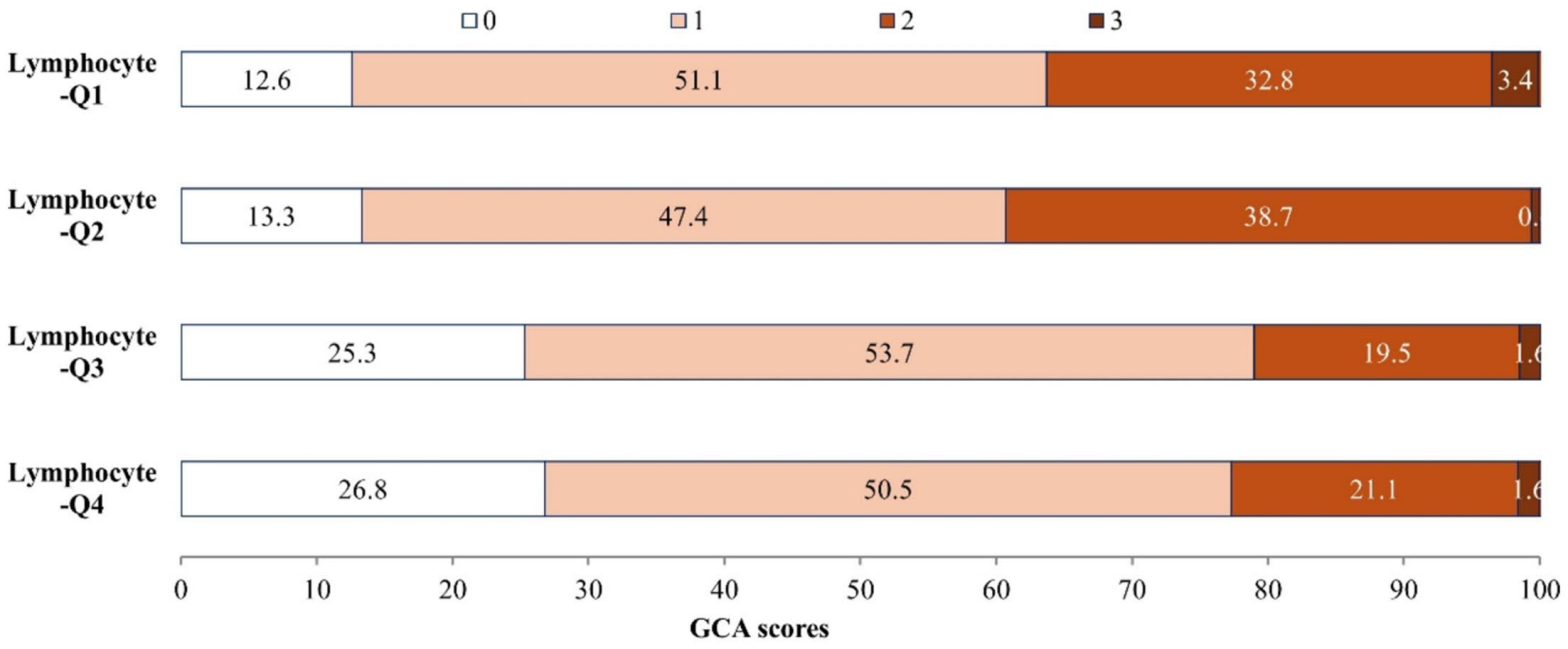
Associations of lymphocyte count with severity of brain atrophy.

There are 583 (80.2%) of 727 AIS patients have brain atrophy. Figure 2 illustrates the distribution of GCA scores across lymphocyte count quartiles. Patients with higher lymphocyte counts demonstrate significantly lower GCA scores (*p* < 0.001). In the unadjusted logistic regression model, the ORs of brain atrophy were significantly lower among study participants with admission lymphocyte count in the highest quartile (Q4 ≥ 2.1*109/L) compared with those in the lowest quartile (Q1 < 1.2* 109/L) (OR 0.39, 95% CI 0.23–0.68) (Table 3). In adjusted model, compared with the lowest quartile, the ORs (95% CIs) for the highest quartile of lymphocyte counts were 0.46 (0.23–0.92) for brain atrophy (Table 3). Additionally, when considering lymphocyte count as a continuous variable, a higher level of lymphocyte count was still significantly associated with decreased odds of brain atrophy (OR 0.68, 95% CI 0.49–0.93) (Table 3). The AUC and *p*-value for baseline lymphocyte count in predicting brain atrophy was 0.615 (95% CI: 0.565–0.666, *p* < 0.001) (Supplementary Figure S1).

**Table 3:** Associations of lymphocyte count with brain atrophy.

Lymphocyte count, 10^9^/L						Continuous of lymphocyte
	Q1: <1.2	Q2: 1.2–1.6	Q3: 1.6–2.1	Q4: ≥2.1	*p* trend	
Median	0.9	1.3	1.8	2.4		
Brain atrophy						
Events, n (%)	152 (87.4)	150 (86.7)	142 (74.7)	139 (73.2)		
Model 1	1.00	0.94 (0.50–1.77)	0.43 (0.25–0.75)	0.39 (0.23–0.68)	<0.001	1.00 (0.96–1.03)
Model 2	1.00	0.91 (0.46–1.83)	0.52 (0.28–0.96)	0.49 (0.26–0.90)	0.006	0.97 (0.92–1.01)
Model 3	1.00	1.05 (0.47–2.33)	0.55 (0.27–1.13)	0.46 (0.23–0.92)	0.007	0.68 (0.49–0.93)

Model 1: unadjusted model.

Model 2: adjusted for age (<69 vs. ≥ 69 years old), sex, current smoking and alcohol drinking.

Model 3: adjusted for sex, age (<69 vs. ≥ 69 years old), current smoking and alcohol drinking, systolic BP, fasting plasma glucose, medical history (hypertension, diabetes mellitus, coronary heart disease, atrial fibrillation, ischemic stroke, intracranial hemorrhage), antihypertension treatment, antiglycemia treatment, fibrinogen, neutrophil count.

### 3.4 Subgroup analysis

We found similar inverse associations between baseline lymphocyte count and brain atrophy by baseline NIHSS (≤3 vs. >3) and stroke subtype (LAA vs. Non-LAA), inverse associations between baseline lymphocyte count and WMH by stroke subtype (Supplementary Table S1). No significant interactions between baseline lymphocyte count and subgroup variables were detected (*P*-interaction ≥0.170 for all). There was a significant interaction between baseline lymphocyte count and baseline NIHSS for the WMH (*p* = 0.003 for interaction). The association between higher baseline lymphocyte count and lower odds of WMH was significant only in those with mild stroke (NIHSS ≤3) (P for trend = 0.001) (Supplementary Table S1).

## 4 Discussion

In this study, we found that a higher lymphocyte count was associated with decreased risk of WMH and brain atrophy in patients with AIS. Compared with the lowest quartile, patients with the highest quartile of lymphocyte count were associated with a 14.2–19.7% odds of WMH, PVH, DWMH, and brain atrophy in AIS patients. Our finding provided new evidence that lymphocyte might play an important role in the development of WMH and brain atrophy in patients with stroke. We also found that the inverse relationship between baseline lymphocyte count and WMH was significant only in patients with mild stroke severity (NIHSS ≤3).

CSVD, a prevalent condition in the aging brain, poses a substantial risk factor for cognitive impairment, dementia, and stroke. However, the etiological bases of CSVD remain controversial. Inflammation plays a crucial role in the development of CSVD (Li et al., 2022; Kong et al., 2024; Li et al., 2024; Wan et al., 2023; Zhang et al., 2022; Evans et al., 2021). Systemic inflammation processes are thought to be closely associated with endothelial dysfunction, blood–brain barrier permeability, and cerebral blood flow autoregulation, which may thus influence the development of CSVD (Wardlaw et al., 2019; Evans et al., 2021). Previous studies have demonstrated that markers of systemic inflammation and vascular inflammation/endothelial dysfunction may be associated with the prevalence, severity, and progression of CSVD (Wan et al., 2023; Zhang et al., 2022). These findings suggest that inflammatory states may contribute to the processes of CSVD and plasma inflammatory markers may offer valuable predictive indicators for CSVD.

Lymphocyte depletion may be a chronic inflammatory. Existing epidemiologic studies have shown that the lymphocyte count was associated with major cardiovascular risk factors and an increased risk of various cardiovascular diseases (Núñez et al., 2011; Rudiger et al., 2006). However, studies on the relationship between lymphocytes and CSVD were fewer. A cohort study of 139 patients with AIS showed that the lymphocyte counts were negatively correlated with the NIHSS score and positively correlated with the Glasgow Outcome Scale score, suggesting that the lymphocyte counts were a useful biomarker for predicting the severity and 30-day poor outcomes after AIS (Zhang et al., 2019). Another study of 151 patients with ischemic stroke (NIHSS ≤3 at admission) found that lymphocyte counts correlated with the severity of white matter injury and lymphocyte counts were an independent protective factor for cognitive function in patients with cerebrovascular (Li et al., 2022). Our study indicated an inverse correlation between lymphocyte counts and the incidence and severity of WMH, PVH, and DWMH in AIS patients. Moreover, the inverse relationship between baseline lymphocyte count and WMH was significant only in patients with mild stroke severity (NIHSS ≤3), which was in line with previous findings (Li et al., 2022). We speculate that this may be because patients with severe stroke (NIHSS >3) tend to have lower lymphocyte counts, which could counteract the association between lymphocytes and WMH. Additionally, the small number of patients with severe stroke in our study may have contributed to this finding.

The exact mechanisms underlying the association between the lymphocyte count and WMH remain unclear. However, given the role of lymphocytes as chronic inflammatory markers and components of adaptive immunity, we propose several plausible hypotheses. First, chronic subclinical inflammation resulting in endothelial dysfunction and blood–brain barrier disruption (Nam et al., 2022; Nam et al., 2017; Jian et al., 2020). Various toxic metabolites into the periventricular spaces, damaging the surrounding neural tissue, and eventually result in white matter lesions (Wardlaw, 2010; Fu and Yan, 2018). Second, chronic hypoperfusion may be involved. We know that chronic systemic inflammation is a well-known mechanism leading to atherosclerosis (Ross, 1999; Imtiaz et al., 2012), which may lead to hypoperfusion and worsening WMH volume progression (Nam et al., 2017). Third, lymphocytes are the key players in adaptive immunity, and promote white matter repair through the secretion of substances such as interleukin-10 (Li et al., 2022; Nam et al., 2017; Rosenberg, 2017). Lymphocyte numbers may decrease (lymphocyte apoptosis) in many chronic stressful situations (Li et al., 2022; Nam et al., 2022; Chuang et al., 2023). Thus, a low lymphocyte count may indicate a high burden of vascular risk factors, and these diseases may in turn exacerbate WMH.

Brain atrophy is a key MRI marker in CSVD and is associated with cognitive performances in CSVD patients (De-Guio et al., 2019). Previous studies have demonstrated that inflammation may be involved in brain atrophy in the elderly population (Satizabal et al., 2012). No study focuses on the association between lymphocyte and brain atrophy. Our study showed that the incidence and severity of brain atrophy were reduced in AIS patients with high lymphocyte counts. The mechanisms underlying the association between lymphocyte count and brain atrophy may involve inflammation-mediated neurodegeneration and immune damage. Neuroinflammatory response can trigger cellular necrosis, apoptosis, and synapse loss (Satizabal et al., 2012). Moreover, neuroinflammation induces brain oxidative stress, further exacerbating cellular death (Jin et al., 2010; Kamat et al., 2014). Previous research has indicated that synaptic loss and neural cell death are important biological mechanisms linking brain atrophy to cognitive decline (Cherbuin et al., 2019). Interleukin-10 secreted by lymphocytes is a critical neuroprotective cytokine regulating neuroinflammation (Westman et al., 2012; Bodhankar et al., 2013). Based on the findings of the study, lymphocytes may be an independent protective factor for brain atrophy.

To the best of our knowledge, this is the first report to investigate lymphocyte count and the link to WMH, PVH, DWMH, and brain atrophy in patients with ischemic stroke. In the present study, rigid quality control procedures were used in data collection. Additionally, important confounders were also collected and controlled in the multivariable adjusted models, so our study could provide a more valid assessment of the effect of lymphocyte count on WMH, PVH, DWMH, and brain atrophy. Our findings suggest an important role for lymphocyte count in the pathogenesis of white matter lesions and brain atrophy.

The present study has some limitations. First, this is a cross-sectional study, and cannot be used to reveal causal relationships. Thus, further prospective studies are needed to confirm our findings. Second, we collected only the lymphocyte counts on the first visit after admission, and the process of WMH and brain atrophy was slow and chronic. As such, we were unable to study the association between lymphocyte count changes and WMH and brain atrophy in the setting of ischemic stroke. Third, the present study enrolled patients with ischemic stroke, so the generalizability of our findings might be a concern.

## 5 Conclusion

Our present study showed that lymphocyte count was independently and negatively associated with WMH, PVH, DWMH and brain atrophy, in patients with ischemic stroke. Moreover, the association between a higher baseline lymphocyte count and lower WMH was significant exclusively in patients with mild stroke. Our findings suggest lymphocyte count may be considered as a biomarker to identify WMH and brain atrophy. Further studies are needed to confirm our findings in patients with acute ischemic stroke and other populations.

## Data Availability

The raw data supporting the conclusions of this article will be made available by the authors, without undue reservation.
