# Supplementary material for: Correlations among lymphocyte count, white matter hyperintensity and brain atrophy in patients with ischemic stroke
Source: Front Aging Neurosci. 2025 Jan 8;16:1492078. doi: 10.3389/fnagi.2024.1492078 (PMC11751001; doi:10.3389/fnagi.2024.1492078)
Supplement: Supplementary file 1 [file Data_Sheet_1.docx]

**ONLINE-ONLY SUPPLEMENT**

**Chenchen Liu, et al. Correlations among lymphocyte count, white matter hyperintensity and brain atrophy in patients with ischemic stroke**

**Contents**

**Supplementary Table S1. ORs (95% CIs) for WMH and brain atrophy by baseline lymphocyte count: subgroups analysis.**

**Supplementary Figure S1. Receiver operating characteristics of baseline lymphocyte count on WMH, PVH, DWMH and brain atrophy in patients with acute stroke.**

**Supplementary Table S1. ORs (95% CIs) for WMH and brain atrophy by baseline lymphocyte count: subgroups analysis.**

|  | **lymphocyte count, 10^9^/L** | | | |  | ***P* interaction** | |
| --- | --- | --- | --- | --- | --- | --- | --- |
|  | **Q1: <1.2** | **Q2:1.2-1.6** | **Q3:1.6-2.1** | **Q4:≥2.1** | ***P* trend** |  |  |
| **WMH** |  |  |  |  |  |  |  |
| NIHSS score | |  |  |  |  | 0.003 |  |
| NIHSS ≤3 | 1.00 | 0.45 (0.19-1.03) | 0.45 (0.20-0.98) | 0.27 (0.13-0.59) | 0.001 |  |  |
| NIHSS >3 | 1.00 | 1.23 (0.48-3.18) | 1.50 (0.55-4.14) | 2.09 (0.72-6.05) | 0.158 |  |  |
| TOAST subgroup | |  |  |  |  | 0.852 |  |
| LAA | 1.00 | 0.98 (0.43-2.24) | 0.87 (0.41-1.87) | 0.56 (0.27-1.18) | 0.099 |  |  |
| Non-LAA | 1.00 | 0.56 (0.21-1.47) | 0.65 (0.25-1.66) | 0.49 (0.20-1.22) | 0.181 |  |  |
| **Brain atrophy** |  |  |  |  |  |  |  |
| NIHSS score |  |  |  |  |  | 0.170 |  |
| NIHSS ≤3 | 1.00 | 1.27 (0.45-3.61) | 1.02 (0.41-2.52) | 0.61 (0.25-1.48) | 0.138 |  |  |
| NIHSS >3 | 1.00 | 0.58 (0.12-2.80) | 0.18 (0.04-0.87) | 0.21 (0.04-1.05) | 0.021 |  |  |
| TOAST subgroup |  |  |  |  |  | 0.217 |  |
| LAA | 1.00 | 0.77 (0.24-2.49) | 0.51 (0.19-1.38) | 0.27 (0.10-0.74) | 0.005 |  |  |
| Non-LAA | 1.00 | 1.38 (0.35-5.36) | 0.44 (0.12-1.63) | 0.98 (0.29-3.33) | 0.565 |  |  |

**Abbreviations** BP: blood pressure; WMH: white matter hyperintensities; LAA: Large-artery atherosclerosis; NIHSS: National Institutes of Health Stroke Scale.

Adjusted for sex, age (<69 vs. ≥ 69 years old), current smoking and alcohol drinking, systolic BP, fasting plasma glucose, medical history (hypertension, diabetes mellitus, coronary heart disease, atrial fibrillation, ischemic stroke, intracranial hemorrhage), antihypertension treatment, antiglycemia treatment, fibrinogen, neutrophil count.

**Supplementary Figure S1. Receiver operating characteristics of baseline lymphocyte count on WMH, PVH, DWMH and brain atrophy in patients with acute stroke.**


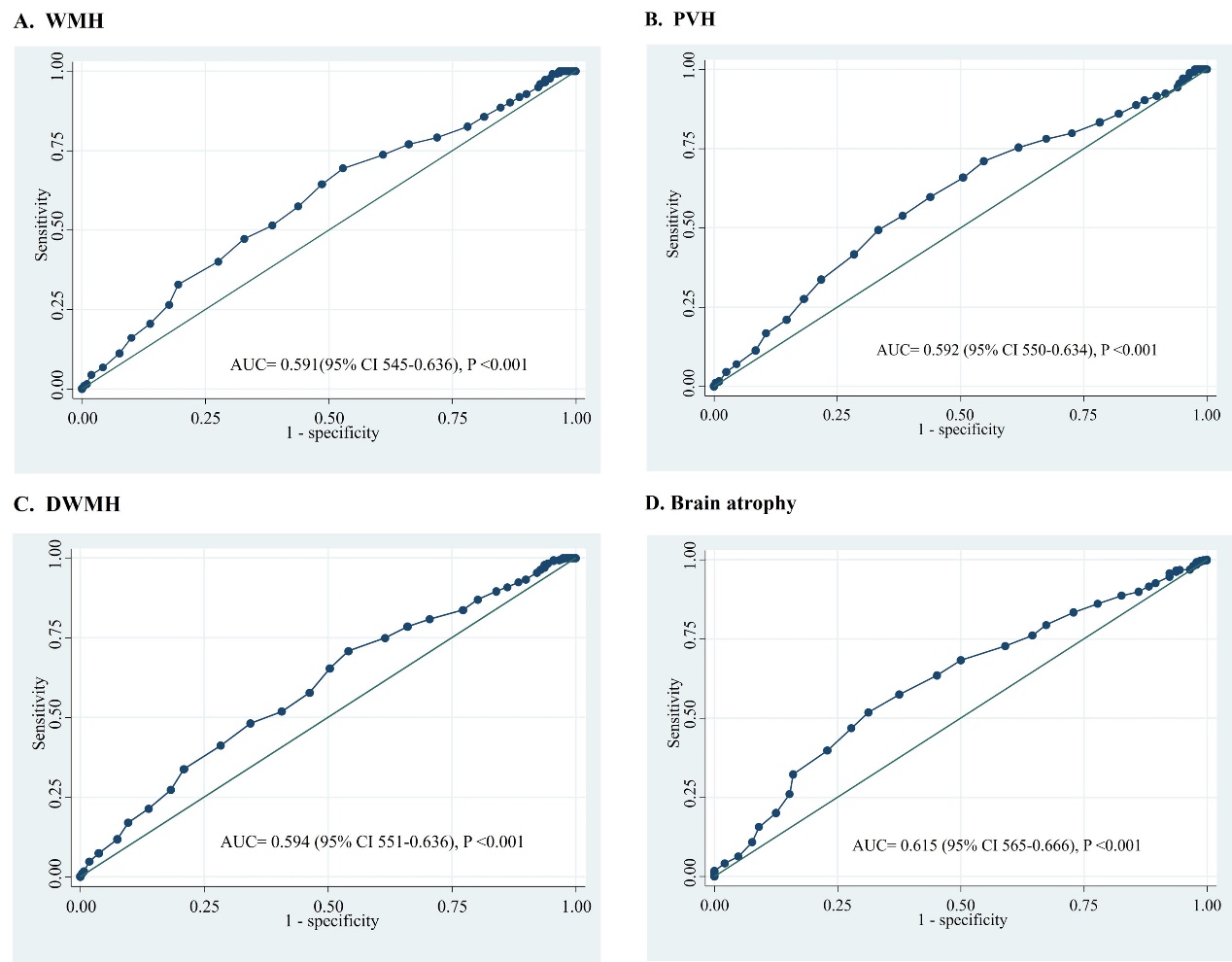


**Abbreviations** AUC: Area under the curve; WMH: white matter hyperintensities; PVH: periventricular hyperintensity; DWMH: deep white matter hyperintensity.
